# Evaluation of Subepithelial Abnormalities of the Appendix by Endoscopic Ultrasound

**DOI:** 10.1155/2009/295379

**Published:** 2009-11-12

**Authors:** Lance T. Uradomo, Peter E. Darwin

**Affiliations:** ^1^Division of Gastroenterology and Liver Diseases, George Washington University School of Medicine and Health Sciences, Washington, DC 20037, USA; ^2^Division of Gastroenterology and Hepatology, University of Maryland School of Medicine, Baltimore, MD 21201, USA

## Abstract

*Background*. The use of through-the-scope (TTS) miniprobe catheter endoscopic ultrasound is a valuable technique for evaluating subepithelial lesions in the proximal colon. Few reports include the evaluation of the appendix by EUS. *Objective*. To describe endoscopic and endosonographic characteristics of subepithelial lesions of the appendix. *Methods*. Retrospective case series in a single academic medical center. Adult patients referred for evaluation of subepithelial lesions of the appendix identified by colonoscopy between April 1, 2003 to February 29, 2008. Data were abstracted from an electronic endoscopic database for all patients undergoing miniprobe endoscopic ultrasound examination of the appendix. Medical records were reviewed for patient followup and outcomes. *Results*. Nine cases were identified. Seven (78%) patients were female. Seven (78%) utilized the 12 MHz miniprobe device and two (22%) used the 20 MHz device. Three mucoceles were described and confirmed by surgical resection. Cases also included one inverted appendix, one gastrointestinal stromal tumor, and one lipoma. In three cases, no abnormality was found. *Conclusions*. EUS evaluation of the appendix is feasible with standard miniprobe devices and may assist in the selection of patients who may benefit from surgical management.

## 1. Background

Endoscopic ultrasound (EUS) is a useful technology for the evaluation of diseases throughout the abdomen and mediastinum. In the colon and rectum it has clearly demonstrated utility in the locoregional staging of rectal carcinoma [1]. EUS (with and without FNA) also successfully identifies subepithelial or extrinsic lesions of the left colon and rectum [2]. For more proximal areas of the colon, forward-viewing echocolonoscopes are commercially available but have not gained widespread utilization. The use of through-the-scope (TTS) miniprobe catheter ultrasound has been shown to capably provide staging information for colonic neoplasms throughout the colon [3–5]. Miniprobe ultrasound is also a valuable technique for evaluating subepithelial lesions in the proximal colon. It has the advantage of deployment through a standard forward-viewing colonoscope which allows easier maneuverability and endoscopic visualization. Although it lacks FNA capability, miniprobe EUS can accurately distinguish intramural from extracolonic lesions. It can also identify the wall layer of origin and the echotexture of the lesion to aid in diagnosis [6]. Pneumatosis cystoides intestinales, gastrointestinal stromal tumors (GISTs), lipomas, mucoceles, endometriosis, and carcinoid tumors have been characterized using miniprobe catheters [7–10]. 

Relatively few reports of evaluation of colonic lesions by EUS miniprobes include examination of the appendix. Kameyama et al. included the description of a single appendiceal mucocele in a series of 46 cases (2.2%) [7]. The present report describes a series of patients referred specifically for evaluation of subepithelial lesions in the appendix identified by colonoscopy.

## 2. Patients and Methods

A single academic center electronic endoscopy database review (Provation, Provation Medical, Minneapolis, MN) was performed for April 1, 2003 to February 29, 2008. A total of 2934 EUS cases were performed during the reviewed time period. Of those, 380 were characterized as lower EUS (13%). Additional cases were identified by searching the database for the use of a miniprobe EUS device. This allowed inclusion of cases that were logged as colonoscopy rather than EUS. Cases were selected if they were referred and evaluated for an appendiceal deformity found on endoscopy or subepithelial tumor versus extrinsic compression of the appendix. Procedure reports and images were reviewed. All cases were performed after a standard bowel preparation and used either a 12 MHz (model UM-2R) or 20 MHz mini probe (model UM-3R; Olympus America, Center Valley, PA) through the instrument channel of an adult or pediatric colonoscope. Visual inspection was followed by filling of the cecal base with sterile water. The miniprobe device was then advanced through the channel of the colonoscope and positioned adjacent to the lesion of interest. A full EUS evaluation was then performed and images captured. 

## 3. Results

### 3.1. Patients and Procedures

Nine procedures were found to meet the inclusion criteria. The procedures were performed by one of two attending gastroenterologists. All but two included the participation of gastroenterology fellows. The cases were performed with moderate sedation using a mean of 131 mcg of fentanyl (range 100–200 mcg) and 3.67 mg of midazolam (range 2–5.5 mg). The mean age of the patients was 58 years (range 50–77). Seven (78%) were female. All were outpatients. Seven of the nine cases (78%) utilized the 12 MHz miniprobe device (Table 1).

### 3.2. Findings

The findings are summarized in Table 1. In two cases, the appendiceal orifice appeared normal by both the endoscopic and endosonographic exams. One case identified periappendiceal erythema and aphthous ulceration, which appeared normal by EUS, and revealed active colitis (mucosal infiltration of inflammatory cells) on histologic examination.

One case was found to be an inverted appendix. The endoscopic suspicion was confirmed by the EUS appearance of concentric rings made up of the mural layers of the appendix. 

**Figure 1:**
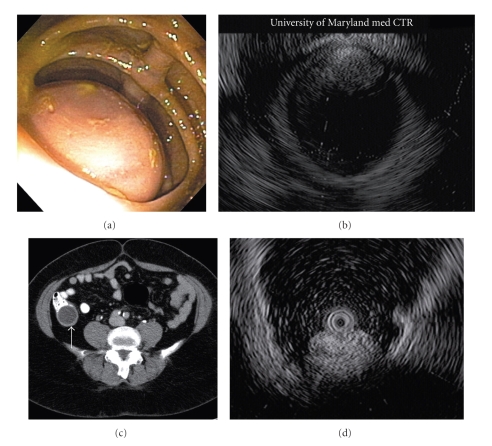
Endoscopic, EUS, and radiographic images of appendiceal findings. (a) Endoscopic image of an appendiceal mucocele; (b) endosonographic image of an appendiceal mucocele; (c) CT scan image of an appendiceal mucocele (arrowhead); (d) endosonographic image of an appendiceal lipoma.

Three patients were referred to surgery based on the findings of the EUS. They each underwent ileocecectomy—one open and two laparoscopically—which removed mucinous cystadenomas, a type of mucocele. By EUS, they were described as anechoic to hypoechoic and heterogenous (Figure 1(b)). In each case, the endosonographer found it difficult to determine the wall layer of origin. 

One case identified a <1 cm subepithelial mass at the appendiceal orifice. The sonographic appearance was hypoechoic, homogenous, and well defined. It appeared to originate from the muscularis mucosa. By EUS, it measured 7 mm by 3 mm in cross-sectional diameter. Biopsies with jumbo forceps were nondiagnostic. The most likely diagnosis was felt to be a GIST. The patient decided not to undergo surgery for this lesion. Followup exam one year later showed it was unchanged. 

Finally, one lesion was described endoscopically as a medium-sized subepithelial mass at the appendiceal orifice. The sonographic appearance was hyperechoic, homogenous, and well defined. It originated within the submucosal layer. Based on these findings, it was felt to be most consistent with a lipoma (Figure 1(d)). No further intervention was recommended.

## 4. Discussion

Complete endoscopic evaluation of the colon involves visualization of the base of the cecum and ostium of the vermiform appendix. Primary mucosal adenoma and adenocarcinoma of the appendiceal orifice may be diagnosed with standard endoscopy and biopsy [11]. However, subepithelial abnormalities of the appendix may pose a diagnostic challenge. We present the largest series to date utilizing miniprobe EUS to help evaluate such lesions.

A bulging or subepithelial lesion of the appendix may have multiple etiologies. In the present series, three appendiceal mucoceles were identified and subsequently referred for surgical management with pathologic confirmation. One lesion had a typical appearance with a 3 cm anechoic lesion with a papillary formation on the wall. Two other lesions were smaller in size (10 mm and 15 mm diameter) and had a hypoechoic and heterogenous appearance without a defined cystic space. Based upon these features, other lesions such as carcinoid could not be excluded preoperatively.

Appendiceal mucoceles may appear as smooth bulbous subepithelial lesions of the cecum with an impression formed by the appendiceal orifice [12]. Mucoceles have variable pathologic features and may have underlying mucinous cystadenomas or cystadenocarcinomas. They have been classified as low-grade mucinous neoplasm, mucinous cystadenocarcinoma, or discordant based on architectural and cytologic features [13]. Rupture and spread of mucin and/or dysplastic epithelium into the abdominal cavity can occur with subsequent pseudomyxoma peritonei and death. Recognition and surgical resection of these lesions is therefore required. Several EUS case reports of this lesion have been reported. These describe an extrinsic cystic mass with papillary, tumor-like elevations [14, 15]. The wall layer of origin was difficult to ascertain for the lesions in the current series. This may be due to their extrinsic location and the limited depth of penetration of miniprobe EUS.

Three patients had normal findings by endoscopy and EUS. These patients were stable and asymptomatic on clinical follow-up ranging from one to four years. An inverted appendiceal orifice—a nonpathologic finding—was seen in one patient in the series. This has been previously described by both virtual and standard colonoscopy [16, 17]. Appendiceal intussusception has also been diagnosed with endoscopic ultrasonography based upon the multiconcentric appearance [18].

One lesion had the sonographic appearance typical of a lipoma. The EUS characteristics of lipomas have been well described throughout the gastrointestinal tract. As for the lesion in this series, the location within the submucosa and its hyperechoic, homogenous appearance was sufficient to confidently make the diagnosis [7]. Based upon its sonographic characteristics, a 3 mm by 7 mm lesion arising from the muscularis mucosa was thought to represent a small GIST. No diagnostic pathology specimen was obtained, and the lesion was unchanged at 1 year follow-up. Mesenchymal tumors of the appendix are very rare, with only four identified from the files of the Armed Forces Institute of Pathology from 1970 to 1998 [19]. 

Appendiceal malignancies are very rare. None were seen in this case series. In a histopathological review of 2154 patients who underwent appendectomy over a 9-year period, 22 were found to have malignant appendiceal neoplasms [20]. These included carcinoid, adenocarcinoma, lymphoma, and metastasis. 

Appendiceal carcinoid tumor most often presents as appendicitis. It is the most common type of appendiceal primary malignancy and is found in 0.3%–0.9% of patients undergoing appendectomy [21]. Goblet cell carcinoma is a rare neoplasm of the appendix that shares features of both adenocarcinoma and carcinoid [22]. The malignant potential is more aggressive than conventional carcinoid. Endoscopic ultrasound is a highly sensitive method for evaluating carcinoid tumors of the stomach, duodenum, and rectum, but there is no literature on evaluation of appendiceal lesions [10, 23].

Our series of patients were referred specifically to evaluate abnormal findings of the appendix identified by colonoscopy. EUS evaluation of the appendix is feasible with standard miniprobe devices and may allow selection of those in need of surgical management. Miniprobe EUS is not technically difficult and may be performed by experienced endosonographers without significant additional training. Several patients in our series had benign findings by endosonographic evaluation and were followed clinically. Others were subsequently referred for surgical resection.

## Figures and Tables

**Table 1:** Patients and findings.

Case	Age	Gender	Device	Endoscopic finding	EUS findings						Surgery	Diagnosis
					Size (mm)	Shape	Echotexture	Homogenity	Definition	Wall layer		
1	56	Male	12 MHz	Normal	No lesion						None	Normal
2	56	Female	12 MHz	Normal	No Lesion						None	Normal
3	55	Female	20 MHz	Erythema and small	No Lesion						None	Colitis
				aphthoid ulcers around								
				appendiceal orifice								
4	77	Male	12 MHz	Inverted appendix	5 × 7	Oval	Concentric	NA	Well defined	NA	None	Inverted
							rings					Appendix
5	50	Female	12 MHz	Submucosal mass	13 × 13	Round	Hypoechoic	Heterogenous	Well defined	Unclear	Laparoscopic right	Mucocele
											hemicolectomy	
6	60	Female	20 MHz	Appendix protuberant and	15 × 15	Round	Hypoechoic	Heterogenous	Well defined	Unclear	Laparoscopic	Mucocele
				prominent							cecectomy	
7	63	Female	12 MHz	Submucosal mass	34 × 30	Oval	Anechoic with	Heterogenous	Well defined	Unclear	Open ileocecectomy	Mucocele
							debris					
8	51	Female	12 MHz	Submucosal mass	7 × 3	Oval	Hypoechoic	Homogenous	Well defined	Muscularis	None	Suspected
										Mucosae		GIST
9	56	Female	12 MHz	Submucosal mass	5 × 5	Round	Hyperechoic	Homogenous	Well defined	Submucosa	None	Lipoma
